# Focal-onset seizure due to left internal carotid artery dissection in the context of confounding hyperglycaemia

**DOI:** 10.1093/omcr/omac028

**Published:** 2022-03-16

**Authors:** Zachary Moulder, Monika Kosela, M Ahtsham Zafar, Abhinav Jha, Karthik Gopal, Anmol Pandey

**Affiliations:** University College London Medical School, London, UK; University College London Medical School, London, UK; Salford Royal NHS Foundation Trust, Salford Royal Hospital, Stott Lane, Salford, UK; St George’s University Hospitals NHS Foundation Trust, St George’s Hospital, London, UK; Quest Teleradiology, JP Nagar, Bengaluru, Karnataka, India; North West Anglia NHS Foundation Trust, Peterborough City Hospital, Peterborough, UK; University College London Hospitals NHS Foundation Trust, The National Hospital for Neurology and Neurosurgery, UCL Queen Square Institute of Neurology, Queen Square, London

## Abstract

A 36-year-old diabetic woman presented to hospital with a seizure that started with shaking of the right hand which sequentially progressed to the entire right side of the body with associated loss of consciousness. Capillary Blood Glucose was 29 mmol/L. HbA1c was 133 mmol/L. Non-contrast computerised tomography (CT) scan of the brain was normal suggesting that the cause of her seizure was hyperglycaemia. However, Magnetic Resonance Imaging (MRI) of the brain showed infarcts in the left paracentral lobule and caudate nucleus. It also identified loss of signal flow void in the intracranial segment of the left internal carotid artery (ICA) raising the suspicion for thrombosis secondary to dissection. This was later confirmed on CT angiogram. This case demonstrates how the initial CT Head was non-diagnostic. We stress the importance of taking a careful seizure history and subsequently obtaining an MRI scan to fully exclude structural pathology.

## INTRODUCTION

The annual incidence of carotid artery dissection is approximately 2.5–3 per 100 000 patients [1]. Causes are either traumatic or atraumatic. Presentation can include seizures, headaches or even focal neurological deficits which can become permanent due to prolonged ischaemia causing cerebral infarcts. We report the case of a 36-year-old Type 2 diabetic woman, initially thought to have had a hyperglycaemic seizure, who instead had cerebral infarcts secondary to a left ICA dissection which was likely the true cause of her seizure. We stress the importance of taking a careful history of the seizure and we caution against the reliance on a CT scan to exclude structural pathology.

## CASE REPORT

A 36-year-old woman presented to the emergency department after a witnessed seizure which resulted in loss of consciousness. In particular, she described shaking of the right hand which progressed sequentially to involve the right arm and then the right leg. She then lost consciousness for approximately 2–3 minutes after which she regained consciousness but remained drowsy. In the preceding 48 hours, she reported that she had two episodes of shaking in her right hand. These lasted approximately 2–3 minutes and resolved spontaneously. She had no previous seizure history. On examination, her Glasgow Coma Scale was 15/15 and she had no neurological deficits as a result of the seizure. She had a 21 year history of Type 2 diabetes mellitus which was managed with oral hypoglycaemic drugs. She had no other significant medical history. Her capillary blood glucose level was 29 mmol/L and her HbA1c level was 133 mmol/L. Her HbA1c had ranged between 114 and 144 mmol/L over the last 6 years indicating poor glycaemic control. Her remaining blood tests were normal. Apart from the patient’s ethnicity (South East Asian) and diabetic status there were no other cardiovascular risk factors for a stroke. A non-contrast CT scan of her brain was normal. A lumbar puncture ruled out intracranial infection as it showed mildly raised cerebrospinal fluid glucose and protein levels. At this point, it was felt that hyperglycaemia was the most likely cause for her seizure. Accordingly, her glycaemic control was optimised and preparations for discharge were made.

**Figure 1:**
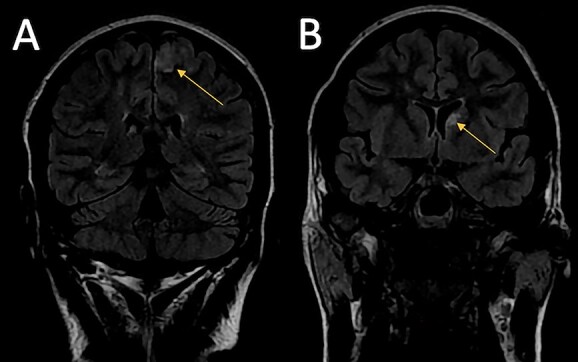
Images A and B are coronal views of the fluid attenuated inversion recovery (FLAIR) MRI sequence of the brain. Image A shows a high signal in the left paracentral lobule. Image B shows a high signal in the left caudate nucleus.

**Figure 2:**
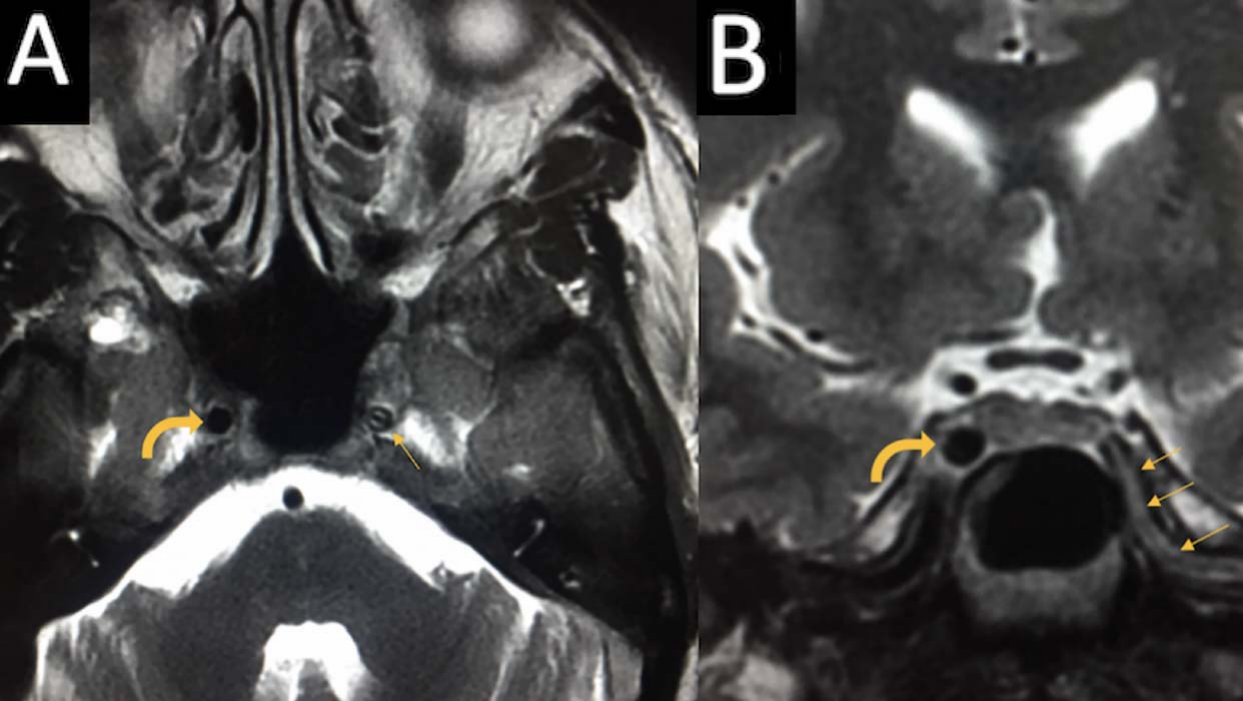
Images A and B are axial and coronal views of the T2 MRI sequence of the brain, respectively. Image A shows that the petrous segment of left ICA (straight arrow) is abnormal with a thin central hypointense signal (black lumen) which is compressed with a crescent of high signal (haematoma) in the vessel wall. In comparison, the right ICA shows a normal signal void (curved arrow). Image B shows that the petrous segment of the left ICA (straight arrows) shows abnormal vessel contour in continuity with a thin linear central hypointensity (black lumen) which is compressed with a high signal (haematoma). In comparison the right ICA shows normal vessel calibre (curved arrow).

**Figure 3:**
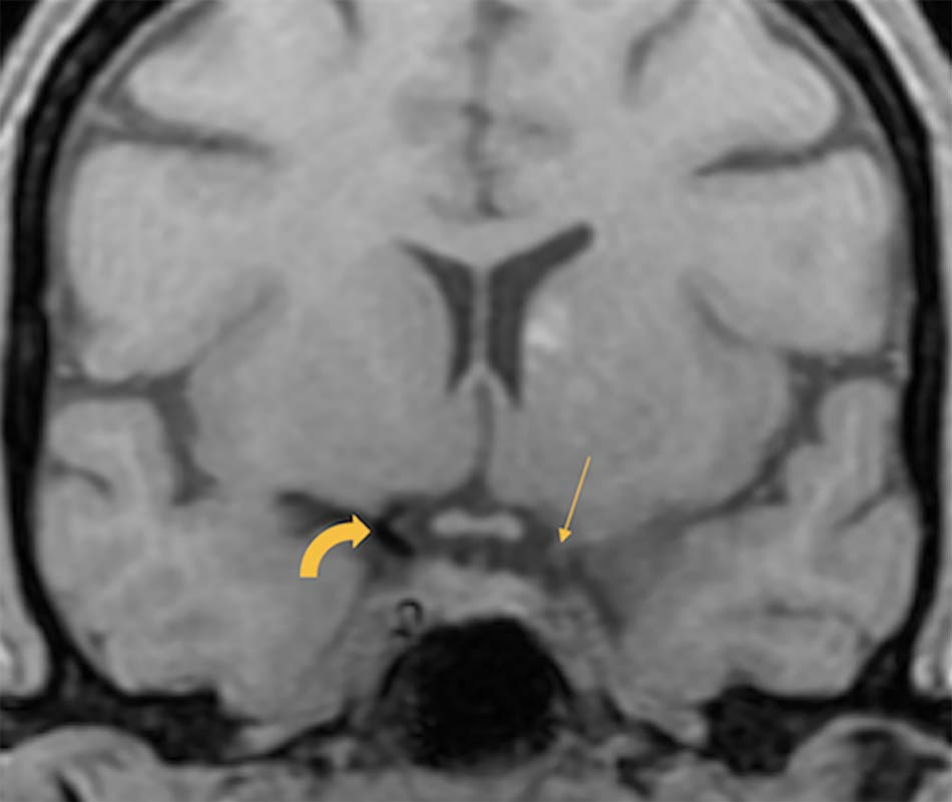
T1 coronal volumetric view of the brain shows loss of the flow void in the left ICA and its thin calibre (straight arrow). In comparison, the right ICA shows normal flow void (curved arrow).

**Figure 4:**
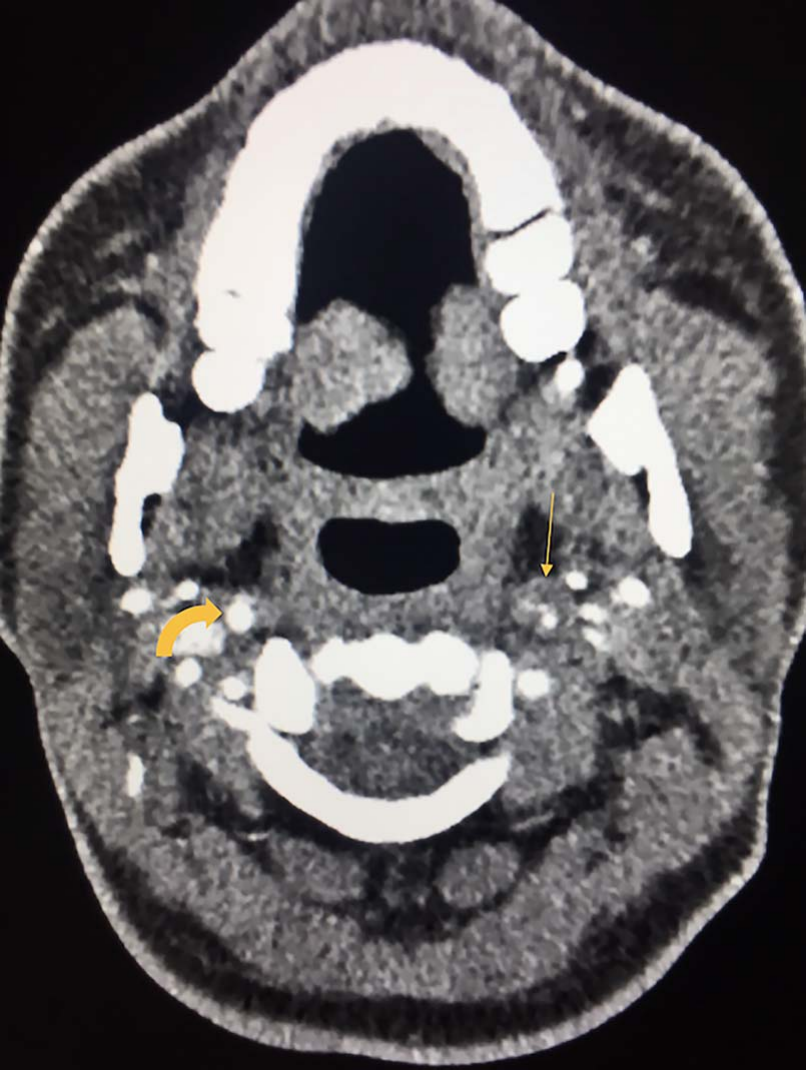
Axial image on CT angiogram shows enlargement of the left ICA diameter with a narrowed eccentric lumen compressed by the approximately isoattenuating intramural hematoma (straight arrow) relative to the surrounding muscle. This is in comparison to the normal enhancement of the right ICA (curved arrow).

**Figure 5:**
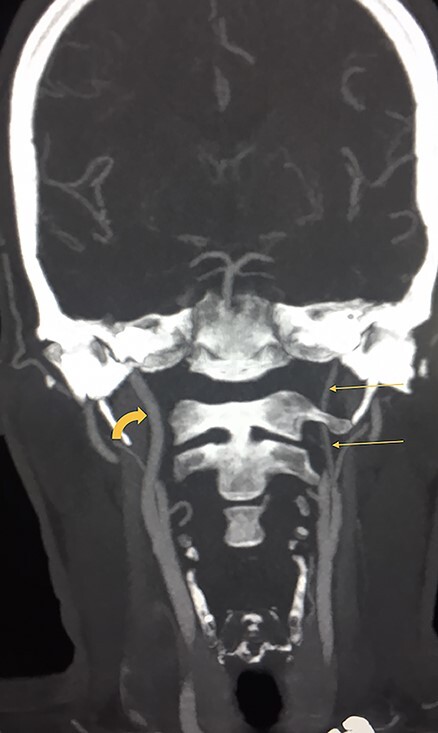
CT angiogram coronal maximum intensity projection (MIP, 0.625 mm slice thickness) reconstruction image shows a long-tapered stenosis in the left ICA, originating beyond the carotid bulb (straight arrows). This is in keeping with a dissection. Normal ICA on the right (curved arrow).

Nonetheless, an MRI scan of the brain was organised to definitively exclude structural pathology. This MRI showed that the patient had a subacute infarction of her left paracentral lobule as well as an infarction of the head of her left caudate nucleus. In addition, the MRI also showed that she had loss of the normal vascular flow void of the intracranial segment of the left ICA raising the suspicion of thrombosis of the vessel secondary to a dissection. Consequently, a CT angiogram was arranged and it confirmed near complete dissection of the left ICA. This pathology is depicted in Figs 1–5.

**Figure 6:**
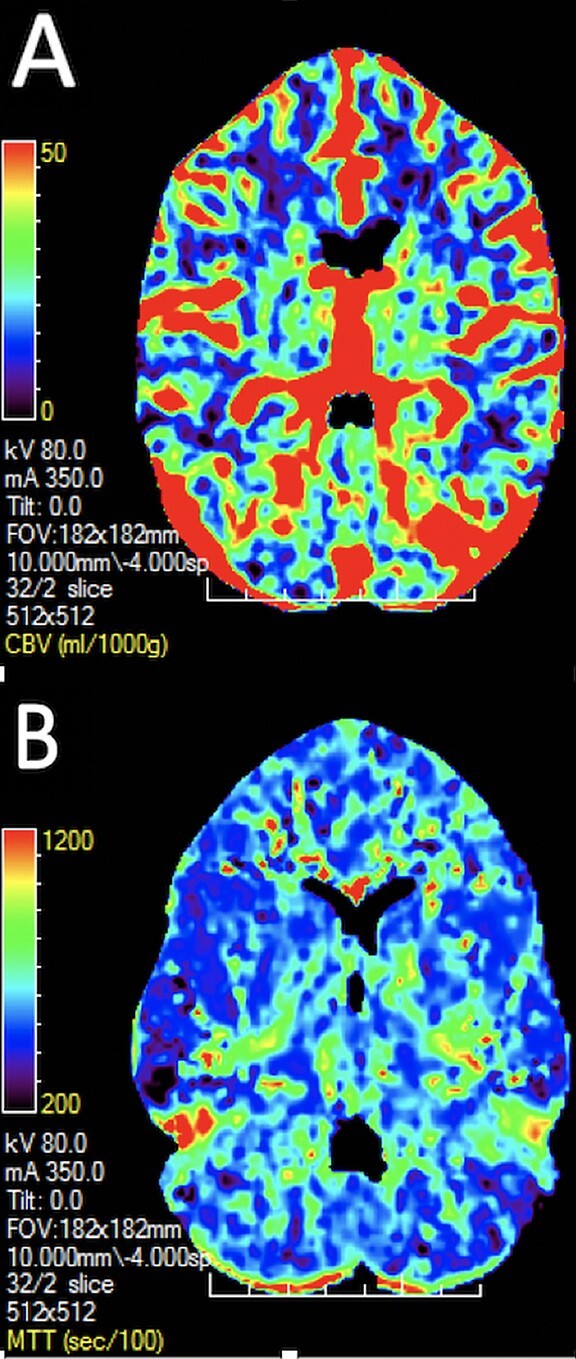
Images A and B show the CT perfusion map appearances of cerebral blood volume and mean transit time, respectively. Image A shows a normal and symmetrical appearance of cerebral blood volume (a measure of the volume of blood (ml) in a given amount of brain tissue (1000g)). Image B shows a normal and symmetrical appearance of mean transit time (a measure of how quickly blood is moving through the brain). Note, in both images, the grey and white matter of the brain return different signals owing to their different perfusion parameters.

**Figure 7:**
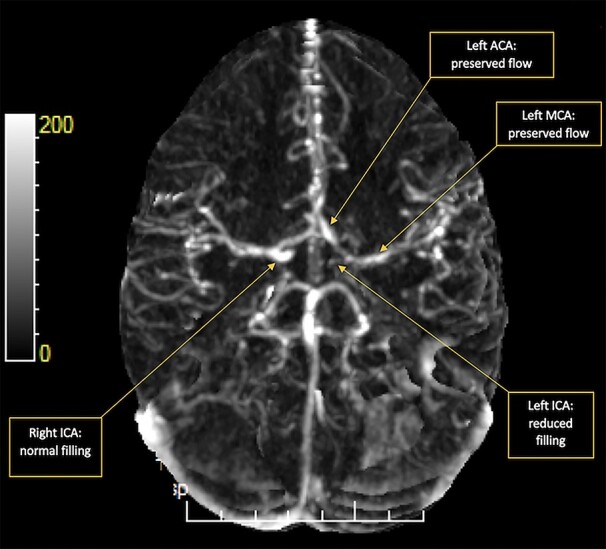
4D MIP Digital Subtraction Angiography image shows normal filling in the distal right ICA but reduced filling in the left ICA with preserved flow in the left proximal ACA and MCA possibly due to interhemispheric flow via the anterior communicating artery.

Subsequent CT perfusion studies demonstrated no significant perfusion defect caused by the ICA dissection (Fig. 6). The derived CT angiogram demonstrated reduced filling in the left ICA. There was preservation of flow in the left middle cerebral artery (MCA) and left anterior cerebral artery (ACA), possibly due to adequate interhemispheric flow via the anterior communicating artery (Fig. 7). She underwent further blood tests for anti-nuclear antibody, antineutrophil cytoplasmic antibody, anti-immunoglobulin G antibody anti-cardiolipin antibodies, all of which were negative. As part of a wider serological work-up, she also had negative results for lyme serology, hepatitis serology, human immunodeficiency virus, treponema pallidum and paraneoplastic antibodies. She was then started on anti-epileptic and anticoagulation therapy as management. She was reviewed via telephone 9 months later, at which point, she reported no further seizures or any other residual symptoms. She mentioned that she was back to her usual quality of life at this time.

## DISCUSSION

A prospective cross-sectional Indian study comprising of 100 patients with adult onset seizures reported that stroke was the aetiology of the seizures in approximately 40% of patients presenting with a focal seizure [2]. Moreover, ICA dissection is known to account for approximately 20% of stokes that occur in patients under 45 years of age [3–5]. Collectively, these results stress the importance of ruling out other causes of focal-onset seizures such as ICA dissection when assessing a patient presenting with a new focal onset seizure even the context of convincing factors such as hyperglycaemia. The involuntary shaking of the right hand followed by sequential progression of seizure activity to the entire right body suggested that the focus of her seizure was the left primary motor cortex. This is well correlated with infarction in her left paracentral lobule given its anatomical proximity to left primary motor cortex. We felt that the left caudate nucleus infarction was likely secondary to seizure activity for two reasons. Firstly, it was of a lower hyperintensity as compared with the infarction in the left paracentral lobule. Secondly, the anatomical location of the left caudate nucleus infarction did not correlate as well with the clinical description of the seizure.

This case also demonstrates how it is insufficient to rely on CT to exclude structural pathology. Recent studies have revealed that CT failed to detect abnormalities which were detected on MRI in 26–57% of cases of a newly presenting seizure [6]. The National Institute for Health and Care Excellence Clinical Guideline [CG137] section 1.6.21 states that MRI `is particularly important' in any patient with a seizure who has `any suggestion of a focal onset on history, examination or [electroencephalogram] (unless clear evidence of benign focal epilepsy)’ [7]. This case serves as a rationale behind the existence of such guidelines and provides some context in which these guidelines should be interpreted. In conclusion, we stress the importance of taking a careful history when evaluating a patient presenting with a new focal-onset seizure and we advise clinicians to always rule out other causes of seizures even in the context of convincing factors.
